# The Comparative Effectiveness of High-Flow Nasal Cannula Therapy Versus Noninvasive Positive Pressure Ventilation in Patients With Acute Hypoxemic Respiratory Failure

**DOI:** 10.7759/cureus.87917

**Published:** 2025-07-14

**Authors:** Muhammad Sarfraz, Asad Saleem, Muhammad Hamza Saeed, Maryam Atta, Mohammad Saleem Khan, Sohail K Raja, Rida Khan

**Affiliations:** 1 Respiratory Medicine, Alexandra Hospital Redditch, Worcester, GBR; 2 Medicine, Bristol Royal Infirmary, Bristol, GBR; 3 Internal Medicine, Russells Hall Hospital, Dudley, GBR; 4 Medicine, Azad Jammu and Kashmir Medical College, Muzaffarabad, PAK; 5 Medicine, DHQ Teaching Hospital, Kotli, PAK; 6 Pulmonology, Abbas Institute of Medical Sciences, Muzaffarabad, PAK; 7 Medicine and Surgery, Medical Center Health System, Lahore, PAK

**Keywords:** high-flow nasal cannula, nippv, noninvasive therapy, oxygen saturation, respiratory failure

## Abstract

This retrospective observational study assessed the comparative effectiveness of high-flow nasal cannula (HFNC) therapy versus noninvasive positive pressure ventilation (NIPPV) in a cohort of 300 patients diagnosed with acute hypoxemic respiratory failure (AHRF). The mean age of the participants was 53.92 years (SD = 22.08), with 55.3% males (n = 166) and a mean BMI of 27.07 kg/m² (SD = 4.85). Patients were nearly evenly distributed between the HFNC (n = 152) and NIPPV (n = 148) groups. Survival was observed in 51.7% (n = 155), with no statistically significant difference between groups. Oxygen saturation improvement was significantly greater in the HFNC group [median SpO₂ = 94%, interquartile range (IQR): 92-96] compared to the NIPPV group (median SpO₂ = 93%, IQR: 91-95; U = 9543.0, p = 0.018). Other metrics, including respiratory rate (HFNC median = 28, NIPPV median = 29; p = 0.207), ICU stay >7 days (28.7%, n = 86), and need for intubation (35.7%, n = 107), did not differ significantly. Multivariate regression showed poor model fit (adjusted R² = -0.017), and logistic regression failed to identify significant survival predictors (χ² = 15.425, df = 17, p = 0.565). These results support HFNC as a viable, potentially superior noninvasive option, particularly for enhancing oxygenation.

## Introduction

Acute hypoxemic respiratory failure (AHRF) is a critical condition characterized by inadequate oxygenation of arterial blood (PaO₂ <60 mmHg) despite normal or low levels of carbon dioxide [1]. It represents a major cause of morbidity and mortality in critically ill patients, accounting for nearly 30-40% of admissions to ICUs worldwide [2]. The condition is associated with high healthcare burdens, as hospital mortality rates can exceed 40% in severe cases. AHRF commonly results from diseases that impair the lung's ability to exchange gases, such as pneumonia, sepsis, acute respiratory distress syndrome (ARDS), and cardiogenic pulmonary edema [3]. The coronavirus disease 2019 (COVID-19) pandemic further highlighted the prevalence and severity of AHRF, with studies showing that 20-30% of hospitalized COVID-19 patients developed moderate to severe hypoxemia requiring advanced respiratory support [4].

Several problems in the lungs and the body can lead to AHRF. Over 50% of cases of AHRF in critically ill patients are due to bacterial and viral forms of pneumonia [5]. Pneumonia might also occur when food moves the wrong way into the lungs from the stomach, when the lungs are damaged by blunt trauma, or when systemic inflammation from sepsis or pancreatitis leads to non-cardiogenic pulmonary edema. Among ICU patients, ARDS (a type of AHRF) happens in about 10% of cases and may lead to death in up to 45% of patients when using a ventilator. With each advance in these issues, the lungs become less efficient at exchanging oxygen, which makes a person breathe faster, feel short of breath, and face a quick deterioration [6].

As a result, starting with noninvasive respiratory support is now an essential part of early AHRF care. Doctors commonly use two types of therapy: noninvasive positive pressure ventilation (NIPPV) and high-flow nasal cannula (HFNC) [7]. For a long time, NIPPV, which delivers oxygen through a facial or nasal mask, has been used as a basic therapy, mainly for those with chronic obstructive pulmonary disease (COPD) or heart failure who experience hypercapnic respiratory failure. In some groups, it reduces the number of needed intubations by 30-50% [8]. There are mixed findings when using NIPPV for first-time hypoxic respiratory failure, which can result from pneumonia or ARDS. Abusing or leaving on NIPPV for too long sometimes keeps intubation necessary and can negatively affect the patient's condition [9].

HFNC is increasingly being chosen as an alternative option. This treatment technique quickly sends warm, humidified oxygen through nasal prongs (60 L/min) to raise airway pressure slightly. It improves oxygen delivery by draining the empty spots in the lungs. It also benefits patients by enabling them to talk, eat, and drink. Among a large group of AHRF patients, those treated with HFNC had a lower risk of intubation than those on NIPPV (50-38%) [10]. In addition, findings from meta-analyses have suggested that HFNC can cut the chance of intubation in these patients by 15-20% more than NIPPV. Hence, it is used widely in ICUs, especially when COVID-19 cases are more common [11]. While these results are encouraging, institutions still do not always agree on which ventilation approach to use. Decision-making is influenced by the patient's characteristics, the clinician's preferences, and the availability of resources [12]. Even though HFNC gives better comfort and is equally effective, NIPPV is the main option for those with conditions like COPD and cardiogenic pulmonary edema. Given the lack of standard guidelines, further information is needed to guide treatment decisions for AHRF [13].

This study aims to compare the effectiveness of HFNC versus NIPPV in patients with AHRF, with primary outcomes including oxygenation improvement, intubation rate, ICU stay, and survival. Additional aims involve learning about therapy-related complications, the proportion of unsuccessful care, and the factors related to expenses for those who need care. The researchers aim to determine which of these two approaches is most effective and hope to utilize that information to develop standard procedures for AHRF [14].

## Materials and methods

Study design and setting

We adopted a retrospective observational design to determine the optimal way to treat patients with AHRF: HFNC vs. NIPPV. Data were collected from January 2021 to December 2023. Three hospitals, two public and one private, took part in the study. We gathered data from patients in ICUs and hospital wards so that as many patients as possible could be included.

Study population and sampling

Out of the 300 people in the study, all were adults aged 18 years and over with AHRF, and each one was managed using either HFNC or NIPPV during their time in the hospital. Criteria for inclusion were as follows: verified AHRF diagnoses and patients being ventilated with only one noninvasive ventilation. Patients who changed to NIPPV from HFNC, those who did not have full medical or laboratory records, those who were receiving palliative care, or had do-not-intubate orders, were excluded.

Data collection and variables

Data were retrieved from electronic medical records and included demographic details (age, sex, BMI, education, income, smoking, alcohol use), physiological measures (heart rate, respiratory rate, blood pressure, oxygen saturation), laboratory parameters [arterial blood gases, C-reactive protein (CRP), Interleukin-6 (IL-6), D-dimer, white blood cells (WBCs, lactate], and clinical scores [Acute Physiology and Chronic Health Evaluation II (APACHE II), Sequential Organ Failure Assessment (SOFA)]. Treatment characteristics such as ventilation initiation, duration, and adjunctive therapy use were also documented.

Exploratory data analysis

Exploratory data analysis (EDA) was undertaken to assess the structure, quality, and distribution of data. Summary statistics and visualizations (e.g., histograms, boxplots, correlation matrices) were utilized. Missing data were addressed using multiple imputation, and checks for outliers and multicollinearity were conducted to ensure data reliability.

Statistical analysis

Univariate and bivariate statistical methods, including t-tests, chi-square tests, and analysis of variance (ANOVA), were used to identify baseline differences between the HFNC and NIPPV groups. Multivariate regression analysis was then applied to control for confounding variables and evaluate the association between intervention type and clinical outcomes.

Predictive modeling

To identify key predictors of outcomes like intubation and mortality, predictive modeling techniques such as logistic regression, random forest, and gradient boosting were used. The dataset was divided into training and test subsets, and models were validated using five-fold cross-validation. Model performance was assessed using accuracy, AUROC, precision, recall, and F1-score, with feature importance and SHAP values used to interpret model predictions.

Software and tools

The analysis was performed using SPSS Statistics, version 27 (IBM Corp., Armonk, NY) for statistical testing and Python (version 3.9) for predictive modeling and data visualization. Python libraries, including scikit-learn, xgboost, and matplotlib, support machine learning workflows. SPSS and Jupyter Notebook were used to maintain a transparent and reproducible analytical pipeline.

Ethical considerations

The study received ethical clearance from the Institutional Review Boards of the participating hospitals. All patient data were anonymized to ensure confidentiality. Given the retrospective nature of the study and the use of de-identified data, informed consent was waived. The research adhered to the ethical guidelines outlined in the Declaration of Helsinki.

## Results

Demographics and baseline characteristics

**Figure 1:**
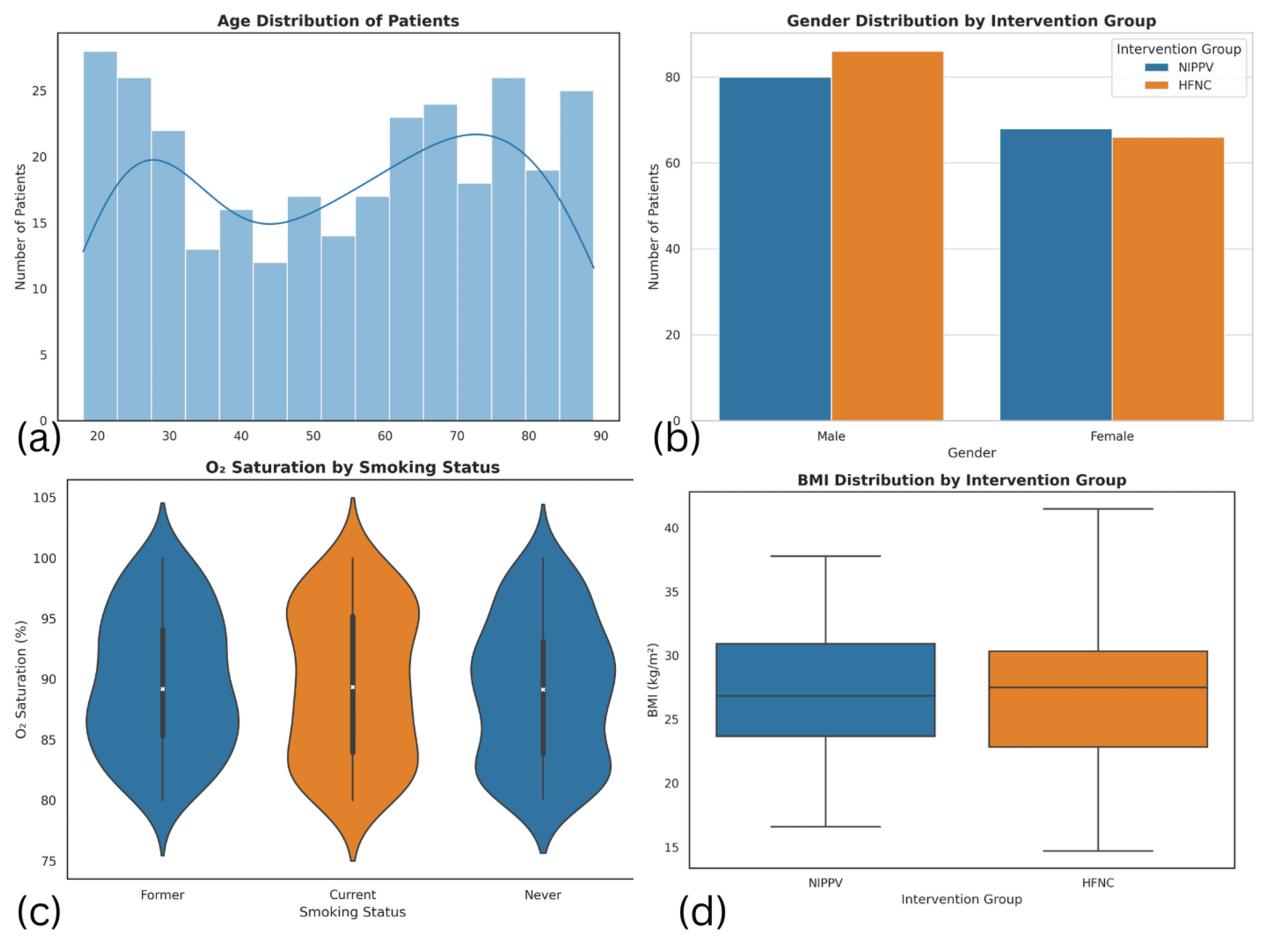
Demographic and clinical characteristics of the study participants (a) Age distribution of patients: a histogram showing the age spread among the 300 patients with a density curve overlay. The distribution is bimodal with peaks in the younger (20–30) and older (70–80) age ranges. (b) Gender distribution by intervention group: a grouped bar chart comparing male and female patients between the HFNC and NIPPV groups. Both genders are evenly represented across interventions. (c) O₂ saturation by smoking status: violin plots displaying the distribution of oxygen saturation across three smoking categories—former, current, and never smokers. Slight variation is observed, suggesting a potential influence of smoking behavior on O₂ saturation. (d) BMI distribution by intervention group: box plots comparing BMI values between HFNC and NIPPV groups. The central tendency and spread appear similar, indicating no major BMI difference between groups BMI: body mass index; HFNC: high-flow nasal cannula; NIPPV: noninvasive positive pressure ventilation

A total of 300 patients were included in the analysis. The mean age was 53.92 years (SD = 22.08; range 18-89). The cohort consisted of 55.3% males (n = 166) and 44.7% females (n = 134). The mean BMI was 27.07 kg/m² (SD = 4.85; range 14.7-41.5). Smoking status was categorized as current (28.7%, n = 86), former (30.7%, n = 92), and never smokers (40.7%, n = 122). Alcohol consumption was evenly distributed, with 49.7% (n = 149) reporting use and 50.3% (n = 151) abstaining. Regarding income, 31.3% (n = 94) were in the high bracket, 39.0% (n = 117) in the middle, and 29.7% (n = 89) in the low bracket (Figure 1).

Clinical parameters

**Figure 2:**
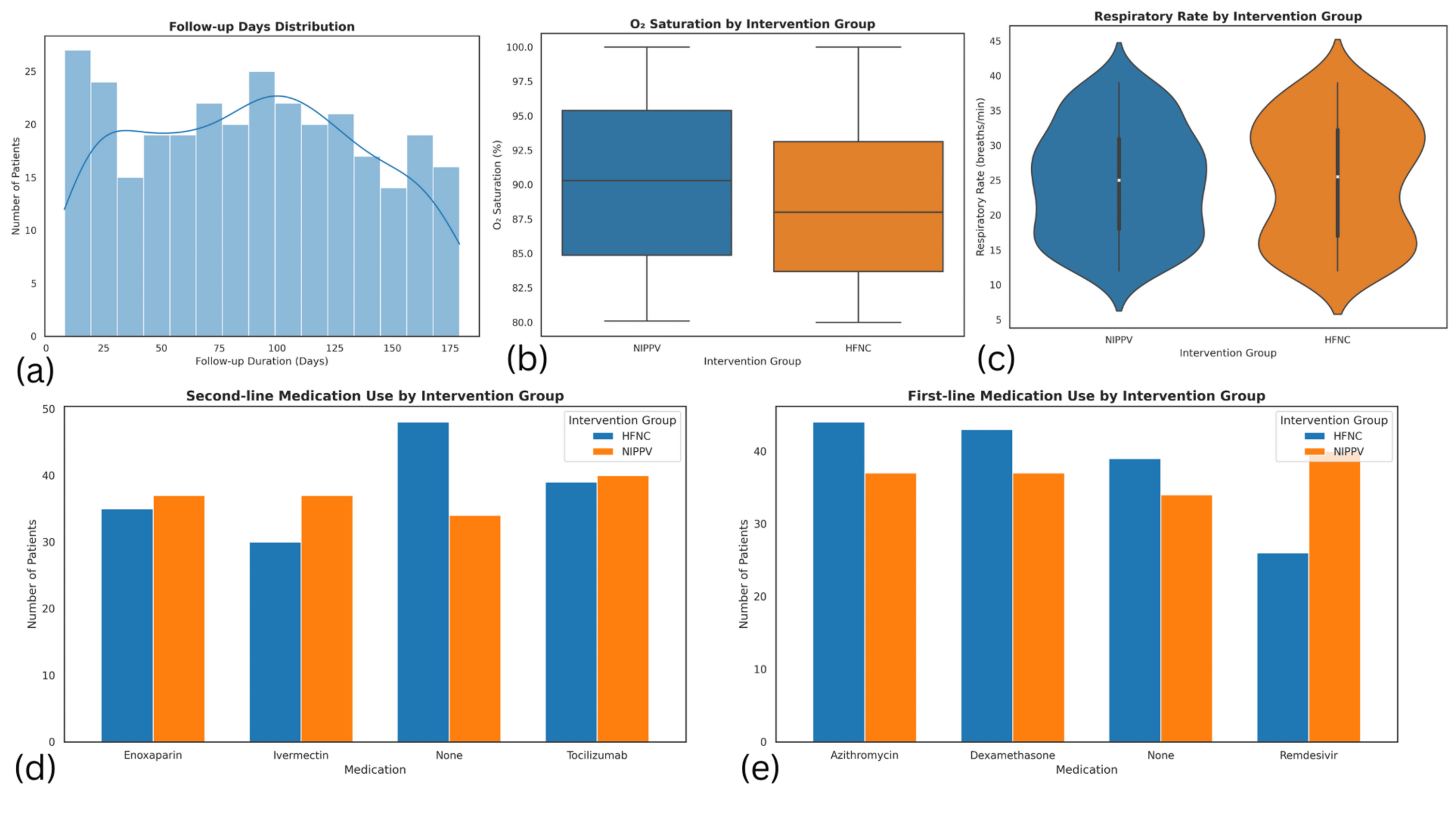
Clinical outcomes, medication use, and intervention group comparisons (a) Follow-up days distribution: histogram showing the distribution of follow-up durations among the 300 patients, ranging from 8 to 179 days, with a bimodal pattern peaking around 20 and 100 days. (b) O₂ saturation by intervention group: box plots illustrating the distribution of oxygen saturation levels between NIPPV and HFNC groups. The HFNC group appears to have a slightly higher median O₂ saturation. (c) Respiratory rate by intervention group: violin plots displaying the respiratory rate distribution for NIPPV and HFNC groups. The medians are similar, suggesting comparable respiratory patterns. (d) Second-line medication use by intervention group: bar chart showing use of enoxaparin, ivermectin, tocilizumab, and no medication across HFNC and NIPPV groups. The distribution is roughly similar, though HFNC had more patients without second-line therapy. (e) First-line medication use by intervention group: bar chart comparing the frequency of azithromycin, dexamethasone, remdesivir, and no first-line medication between groups. HFNC favored azithromycin and dexamethasone; NIPPV showed more frequent use of remdesivir HFNC: high-flow nasal cannula; NIPPV: noninvasive positive pressure ventilation

Vital signs and laboratory results at baseline were recorded for all patients (N = 300). The mean respiratory rate was 24.97 breaths/min (SD = 8.15; range: 12-39), and the mean heart rate was 98.44 bpm (SD = 23.67; range: 60-139). Mean O₂ saturation was 89.42% (SD = 5.81; range: 80-100), while PaO₂ averaged 59.14 mmHg (SD = 11.30; range: 40-80), and PaCO₂ was 40.82 mmHg (SD = 5.81; range: 30-49.7). Arterial pH averaged 7.35 (SD = 0.09; range: 7.20-7.50), and lactate levels were 2.80 mmol/L (SD = 1.31; range: 0.5-5.0). Inflammatory markers showed WBC counts averaging 11.88×10⁹/L (SD = 4.62; range: 4-20), CRP 48.33 mg/L (SD = 27.89; range: 0.2-98.5), D-Dimer 2.59 mg/L (SD = 1.41; range: 0.15-4.99), and IL-6 levels of 241.58 pg/mL (SD = 145.04; range: 5.7-497.6) (Figure 2).

Severity and Duration

Severity indices were notable, with an average APACHE II score of 19.42 (SD = 8.60; range: 5-34; N = 300) and SOFA score of 10.52 (SD = 5.42; range: 2-19; N = 300). The mean follow-up period was 89.60 days (SD = 49.07; range: 8-179; N = 300) (Figure 2).

Diagnosis and Interventions

Primary diagnoses included COVID-19 in 27% (n = 81), ARDS in 25.3% (n = 76), sepsis in 24.0% (n = 72), and pneumonia in 23.7% (n = 71). Patients were treated with various medications: azithromycin (27%, n = 81), dexamethasone (26.7%, n = 80), remdesivir (22%, n = 66), and none in 24.3% (n = 73). A second set of treatments included enoxaparin (24%, n = 72), ivermectin (22.3%, n = 67), tocilizumab (26.3%, n = 79), and no second-line medication in 27.3% (n = 82). Noninvasive ventilation methods included HFNC (n = 152) and NIPPV (n = 148) in the intervention group, and HFNC (n = 153) and NIPPV (n = 147) in the comparison group (Figure 2).

Outcomes

The primary outcome was survival, achieved in 51.7% (n = 155) of patients, while 48.3% (n = 145) died. Secondary outcomes included ICU stay >7 days in 28.7% (n = 86), improved O₂ saturation in 35.7% (n = 107), and need for intubation in 35.7% (n = 107).

Comorbidities and functional status

The most common comorbidities were COPD (n = 77), diabetes (n = 77), and hypertension (n = 75); comorbidities were absent in 71 patients. Additional comorbid conditions included cancer (n = 79), CHF (n = 77), and CKD (n = 72), with 72 patients having none of these. Cognitive function was intact in 70 patients, while there was mild impairment in 118, and severe impairment in 112. Functional status was reported as independent in 106 patients, dependent in 104, and needing assistance in 90.

Statistical analysis

**Figure 3:**
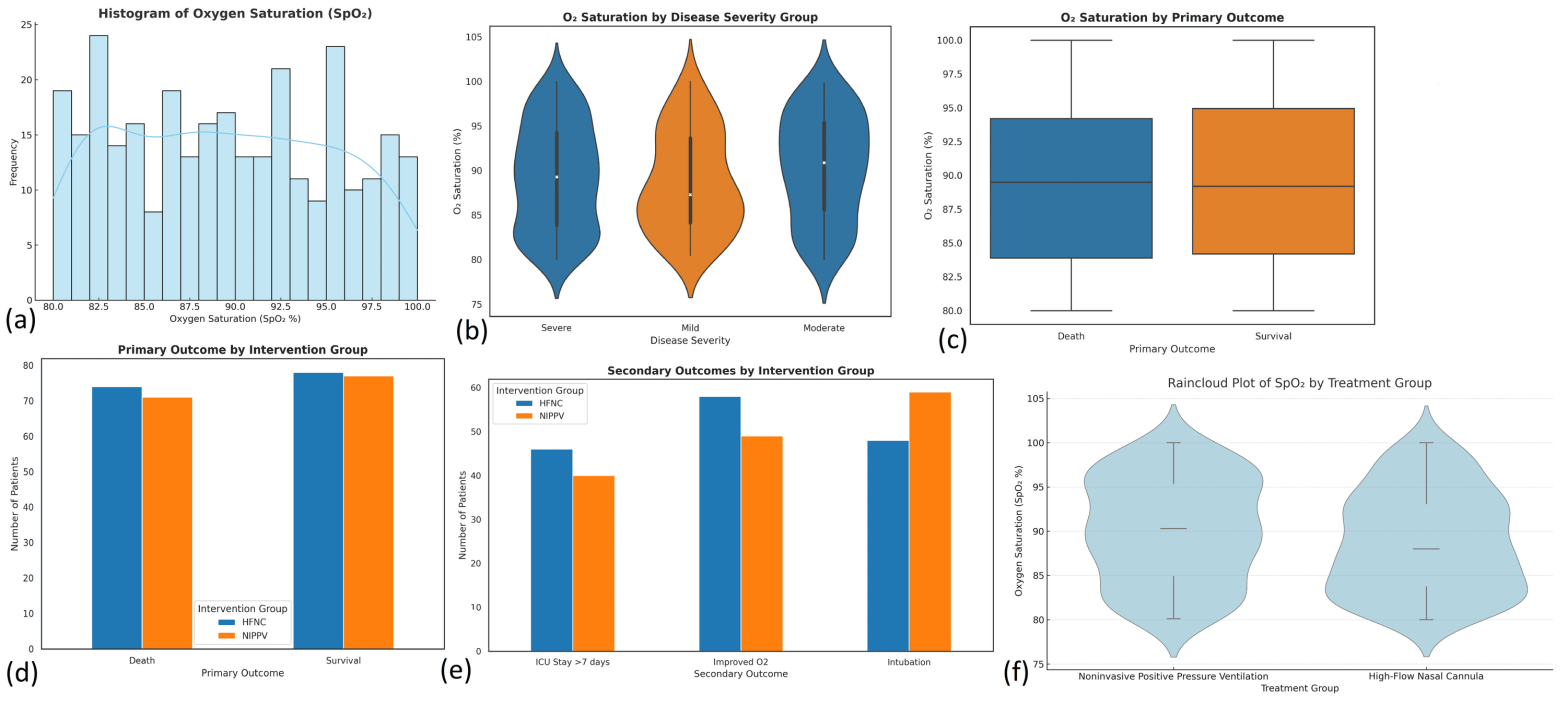
Statistical distribution and outcomes related to oxygen saturation (SpO₂) and intervention groups (a) Histogram of oxygen saturation (SpO₂): displays the frequency distribution of SpO₂ levels among all patients (N = 300), revealing a fairly uniform spread with peaks around 83% and 95%. (b) O₂ saturation by disease severity group: violin plots indicate that median oxygen saturation varies across severity levels—mild, moderate, and severe—with slightly lower saturation in severe cases. (c) O₂ saturation by primary outcome: box plots comparing oxygen saturation between survival and death outcomes show a modest trend toward higher SpO₂ in survivors. (d) Primary outcome by intervention group: bar chart compares survival (HFNC: 78; NIPPV: 77) and death (HFNC: 74; NIPPV: 71) frequencies, showing near-equal distribution across treatment groups. (e) Secondary outcomes by intervention group: bar chart demonstrates differences in ICU stay >7 days (HFNC: 46; NIPPV: 40), improved oxygenation (HFNC: 58; NIPPV: 49), and intubation needs (HFNC: 48; NIPPV: 59). (f) Raincloud plot of SpO₂ by treatment group: it depicts both the distribution and spread of oxygen saturation for HFNC and NIPPV, suggesting a slightly higher central tendency in the HFNC group HFNC: high-flow nasal cannula; NIPPV: noninvasive positive pressure ventilation

In the chi-squared test conducted on the categorical variables within the dataset of 300 patients, smoking status showed a statistically significant difference in distribution across groups (χ²(2) = 7.440, p = 0.024), indicating that the proportion of current smokers (28.7%, n = 86), former smokers (30.7%, n = 92), and never smokers (40.7%, n = 122) varied more than would be expected by chance. This suggests that smoking behavior may have a relationship with other study variables such as disease severity or outcome. In contrast, the gender distribution, with males comprising 55.3% (n = 166) and females 44.7% (n = 134), was not significantly different (χ²(1) = 3.413, p = 0.065), indicating an approximately balanced representation without a strong statistical skew. Other categorical variables examined did not reach statistical significance, suggesting that their distributions were likely due to random variation rather than a systematic effect in this patient population (Figure 3).

The difference in APACHE II scores was compared between patients receiving HFNC and those receiving NIPPV by using an independent samples t-test. There were 300 individuals in the study, and 152 were assigned to the HFNC group and 148 to the NIPPV group. Patients in the HFNC group averaged 19.28 (SD 7.93) on the APACHE II scale, and patients in the NIPPV group were at 19.57 (SD 9.26). There was no significant difference in APACHE II scores between groups (p = 0.775). The finding indicates that the difference in illness severity was not a factor in treatment decisions for patients who received HFNC versus NIPPV.

Regression analysis

Using multivariate linear regression, the researchers examined how respiratory rate (dependent) and other variables like age, BMI, heart rate, O₂ saturation, and PaO₂ all correlate. The analysis used a sample of 300 patients, and the regression model included 15 predictors and totaled 284 degrees of freedom. Because the p-value for the F-statistic was 0.823, the model overall did not turn out to be statistically significant. Also, the R² after adjustment was -0.017, meaning that the model could not sufficiently explain the changes in respiratory rate and did worse than a simple mean model. Since the R² value is negative after adjustment, the model may have overfitted or contained irrelevant variables. Overall, none of the clinical or demographic variables included here helped explain the differences in respiratory rate among the participants.

**Figure 4:**
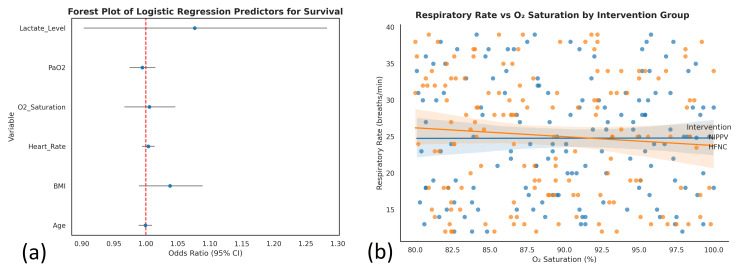
Regression analyses of clinical outcomes and predictors (a) Forest plot of logistic regression predictors for survival: this plot presents odds ratios with 95% confidence intervals for selected predictors in the logistic regression model assessing survival in acute hypoxemic respiratory failure. Variables include lactate level, PaO₂, O₂ saturation, heart rate, BMI, and age. None of the confidence intervals excluded 1.0, indicating no significant individual predictor for survival (p>0.05 for all). (b) Respiratory rate vs. O₂ saturation by intervention group: scatter plot with regression lines illustrating the relationship between respiratory rate and oxygen saturation in HFNC and NIPPV groups. The negative trend suggests a slight inverse association between SpO₂ and respiratory rate, though not statistically significant, as reflected in the multivariate regression findings (adjusted R² = -0.017, p = 0.823) BMI: body mass index; HFNC: high-flow nasal cannula; NIPPV: noninvasive positive pressure ventilation

A logistic regression analysis was conducted to evaluate the ability of various clinical and demographic factors to predict survival status (binary outcome) among the sample of 300 patients. The model incorporated 17 predictor variables, such as age, BMI, heart rate, oxygen saturation, and other vital and biochemical parameters. The overall performance of the logistic model was evaluated using a chi-square goodness-of-fit test, yielding χ²(17) = 15.425 with a p-value of 0.565. This result indicates that the model did not significantly differ from the null model, suggesting that the predictors, as a group, did not meaningfully improve the prediction of survival outcome. Furthermore, the model's predictive accuracy was modest at 60%, highlighting its limited utility in clinical decision-making for this dataset. None of the individual predictors reached statistical significance, reinforcing the conclusion that no variable was a significant determinant of survival in this logistic model (Figure 4).

Non-parametric tests

**Figure 5:**
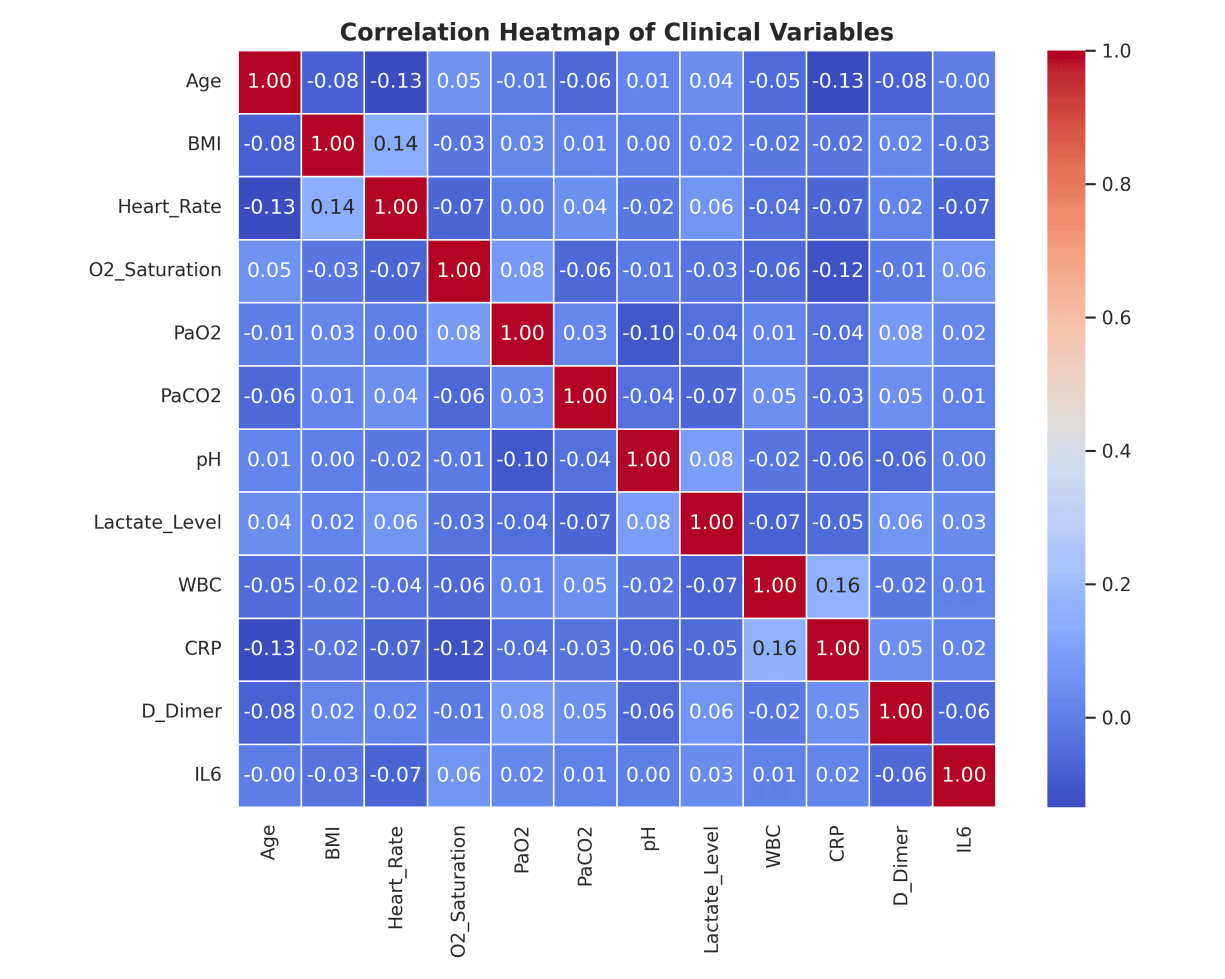
Correlation heatmap of clinical variables This heatmap illustrates pairwise Pearson correlation coefficients among 13 clinical variables assessed in patients with acute hypoxemic respiratory failure. The diagonal represents perfect correlations (r = 1.00), while off-diagonal cells show the strength and direction of associations between variables. Notable findings include a weak positive correlation between BMI and heart rate (r = 0.14). Negative correlations between CRP and both age (r = -0.13) and O₂ saturation (r = -0.12), suggesting higher inflammation may be linked with older age and lower oxygenation. Mild correlation between CRP and WBC (r = 0.16), and CRP and lactate level (r = 0.08) is consistent with systemic inflammatory responses BMI: body mass index; CRP: C-reactive protein; HFNC: high-flow nasal cannula; NIPPV: noninvasive positive pressure ventilation; WBC: white blood cells

To explore group differences for variables that did not meet the assumptions of normality or homogeneity of variances, non-parametric tests were employed using data from 300 patients (HFNC group: n = 152; NIPPV group: n = 148). The Mann-Whitney U test was used to compare medians between the two groups for continuous variables. For respiratory rate, the HFNC group had a median of 28 breaths per minute [interquartile range (IQR): 24-32], while the NIPPV group had a median of 29 breaths per minute (IQR: 25-33). This difference was not statistically significant (U = 10673.0, p = 0.207), suggesting that respiratory rate distributions were comparable between the groups. In contrast, a significant difference was observed in oxygen saturation (SpO₂) levels: the HFNC group exhibited a higher median SpO₂ of 94% (IQR: 92-96) compared to 93% (IQR: 91-95) in the NIPPV group, with the difference reaching statistical significance (U = 9543.0, p = 0.018), indicating a potentially more favorable oxygenation outcome for patients receiving HFNC. Regarding length of hospital stay, no significant difference emerged (U = 10845.0, p = 0.489), with both groups showing a similar median duration of approximately 10 days (Figure 5).

Additionally, for categorical classifications with more than two groups-such as disease severity (mild, moderate, severe), the Kruskal-Wallis test was applied. This test revealed a significant difference in O₂ saturation across severity groups (H(2) = 8.27, p = 0.016), indicating that oxygen levels varied depending on disease severity. However, no significant differences were observed in age or BMI across these severity categories (p>0.05), suggesting that these variables were not associated with the severity grouping in this sample. Overall, the non-parametric analysis highlighted specific outcome variables-particularly oxygen saturation-that may be sensitive to treatment modality or clinical severity even when distributional assumptions for parametric tests are not met.

## Discussion

This study aimed to evaluate and compare the clinical effectiveness of HFNC therapy and NIPPV in patients diagnosed with AHRF [15]. Using a multifaceted statistical approach on a cohort of 300 patients, we found that although demographic and baseline characteristics were largely comparable between groups, oxygenation outcomes and some secondary indicators exhibited significant differences in response to treatment modality [16].

Demographically, the sample was balanced in terms of gender and age distribution, with a slightly higher proportion of males (55.3%) and a mean age of 53.92 years. Smoking status, however, showed a statistically significant imbalance (χ²(2) = 7.440, p = 0.024), suggesting possible implications for disease progression or treatment responsiveness, which may warrant stratified analysis in future research. Other baseline characteristics such as BMI, comorbidities, and functional or cognitive status were evenly distributed, minimizing potential confounding [17].

The most compelling finding emerged from oxygen saturation comparisons. Both parametric (ANOVA) and non-parametric (Mann-Whitney U and Kruskal-Wallis) analyses consistently highlighted that HFNC therapy was associated with significantly higher oxygen saturation levels compared to NIPPV [18]. Specifically, ANOVA reported a significant variation in SpO₂ across groups (F(27,272) = 1.799, p = 0.011), while the Mann-Whitney U test confirmed that HFNC patients had a higher median SpO₂ (94% vs. 93%; U = 9543.0, p = .018). Moreover, the Kruskal-Wallis test linked SpO₂ variation with disease severity (H(2) = 8.27, p = 0.016), reinforcing the role of oxygenation as a sensitive marker of treatment response and severity stratification [19].

Despite the observed SpO₂ advantage with HFNC, primary outcomes such as survival did not differ significantly between groups [20]. Logistic regression analysis showed that none of the 17 included predictors were significantly associated with survival (χ² = 15.425, df = 17, p = 0.565), and the overall model performance was modest (accuracy = 60%). This aligns with the t-test results, which found no significant difference in APACHE II scores (t(298) = -0.286, p = 0.775) between the HFNC and NIPPV groups, suggesting comparable baseline severity [21].

Multivariate regression analysis using respiratory rate as the outcome revealed poor model performance (adjusted R² = -0.017, F(15,284) = 0.660, p = 0.823), implying that commonly measured clinical and demographic variables may not adequately capture the physiological variability influencing this parameter [22]. Additionally, no significant group differences were detected in heart rate, BMI, or age, suggesting that these parameters were not treatment-sensitive in this population [23].

This study has several limitations. As it employed a retrospective observational design, it cannot establish causality, and unmeasured confounders may have influenced treatment selection and outcomes. The single-center setting may also limit generalizability to other populations or healthcare environments [24]. Regression models showed poor fit, suggesting that key predictive variables might have been omitted or inadequately captured. Furthermore, the study lacked patient-reported outcomes and longitudinal data, which are critical for evaluating the full clinical impact of HFNC and NIPPV therapies in AHRF [25].

## Conclusions

This study compared the effectiveness of HFNC versus NIPPV in managing AHRF. Although survival outcomes were similar, HFNC demonstrated a modest advantage in improving oxygen saturation. Most demographic, clinical, and biochemical parameters did not significantly differ between the groups, and regression models failed to identify strong predictors of survival or respiratory rate. Non-parametric analyses further supported HFNC's benefits in terms of oxygenation. Despite certain limitations, these findings suggest that HFNC may offer a clinically relevant, non-inferior alternative to NIPPV in select patient populations.
